# Fundamental movement skills and physical activity among children living in low-income communities: a cross-sectional study

**DOI:** 10.1186/1479-5868-11-49

**Published:** 2014-04-08

**Authors:** Kristen E Cohen, Philip J Morgan, Ronald C Plotnikoff, Robin Callister, David R Lubans

**Affiliations:** 1 Priority Research Centre in Physical Activity and Nutrition, School of Education, University of Newcastle, Callaghan Campus, University Drive, Callaghan NSW, Australia; 2 Priority Research Centre in Physical Activity and Nutrition, School of Biomedical Sciences & Pharmacy, University of Newcastle, Callaghan Campus, University Drive, Callaghan NSW, Australia

**Keywords:** School, Object-control, Locomotor, Lunchtime, Recess, After-school

## Abstract

**Background:**

Although previous studies have demonstrated that children with high levels of fundamental movement skill competency are more active throughout the day, little is known regarding children’s fundamental movement skill competency and their physical activity during key time periods of the school day (i.e., lunchtime, recess and after-school). The purpose of this study was to examine the associations between fundamental movement skill competency and objectively measured moderate-to-vigorous physical activity (MVPA) throughout the school day among children attending primary schools in low-income communities.

**Methods:**

Eight primary schools from low-income communities and 460 children (8.5 ± 0.6 years, 54% girls) were involved in the study. Children’s fundamental movement skill competency (TGMD-2; 6 locomotor and 6 object-control skills), objectively measured physical activity (ActiGraph GT3X and GT3X + accelerometers), height, weight and demographics were assessed. Multilevel linear mixed models were used to assess the cross-sectional associations between fundamental movement skills and MVPA.

**Results:**

After adjusting for age, sex, BMI and socio-economic status, locomotor skill competency was positively associated with total (*P* = 0.002, *r* = 0.15) and after-school (*P* = 0.014, *r* = 0.13) MVPA. Object-control skill competency was positively associated with total (*P* < 0.001, *r* = 0.20), lunchtime (*P* = 0.03, *r* = 0.10), recess (*P* = 0.006, *r* = 0.11) and after-school (*P* = 0.022, *r* = 0.13) MVPA.

**Conclusions:**

Object-control skill competency appears to be a better predictor of children’s MVPA during school-based physical activity opportunities than locomotor skill competency. Improving fundamental movement skill competency, particularly object-control skills, may contribute to increased levels of children’s MVPA throughout the day.

**Trial registration:**

Australian New Zealand Clinical Trials Registry No: ACTRN12611001080910.

## Background

Participation in physical activity is vital for enhancing children’s physical, social, cognitive and psychological development [1]. Higher levels of physical activity in children are associated with improved fitness (both cardio-respiratory fitness and muscular strength) [2], enhanced bone health and reduced body fat [1]. Furthermore, children who regularly participate in physical activity have reduced symptoms of anxiety and depression, and improved self-esteem and confidence [1]. However, the number of children not participating in adequate amounts of physical activity to accrue the associated health benefits is a global concern [3]. Current estimates suggest that only 40% of Australian primary school-aged children are meeting the current physical activity guidelines of 60 minutes of moderate-to-vigorous physical activity (MVPA) every day [4]. Moreover, children from low socio-economic backgrounds are significantly less active than those of middle and high socio-economic backgrounds [4,5].

Schools play a crucial role in providing opportunities for children to be physically active, as they have the necessary equipment, personnel, facilities and curriculum to promote activity [6,7]. Beyond physical education and school sport, lunchtime and recess periods (break time) offer ideal opportunities for children to be active on a daily basis [8]. If provided the choice to be active, the combined lunchtime and recess periods has the potential to contribute up to 40% towards children’s daily physical activity recommendations [8]. Furthermore, the after-school period has been identified as a “critical window” for physical activity [9]. It is a unique period of time where children generally have the discretion to choose their own activities and if engaged in active pursuits, can contribute to approximately 25% of their daily physical activity [10]. It is therefore important to investigate potential factors that are associated with physical activity during key time periods for developing targeted physical activity interventions for children.

Fundamental movement skill (FMS) are considered the building blocks for movement and provide the foundation for specialized and sport-specific movement skills required for participation in a variety of physical activities. FMS can be categorized as locomotor (e.g., run, hop, jump, leap), object-control (e.g., throw, catch, kick, strike), and stability (e.g., static balance) skills [11]. Mastery of FMS is low among Australian primary school-aged children [12], and those from disadvantaged backgrounds often demonstrate the lowest levels of competency [5]. It is suggested that higher levels of FMS competency will provide greater opportunities for children to engage in a variety of physical activities, games and sports. In turn, children who are more skilled will choose higher levels of physical activity, while children who are less skilled will select lower levels of physical activity [13].

A recent systematic review of the health benefits associated with FMS competency found strong evidence for a positive association between FMS competency and physical activity in children [14], but also noted that the majority of studies had used self-report measures of physical activity [15,16]. Such measures are limited by children’s ability to accurately recall their behaviors and generally have low levels of validity and reliability in youth populations [17]. An additional concern is that few studies have adjusted their analyses for weight status, which may moderate the association between physical activity and motor skill proficiency [14,18]. The associations between FMS competency and weight status has been well established, with FMS competency being inversely associated with weight status in children [14,18]. Moreover, children who have a higher weight status participate in significantly lower amounts of MVPA [19].

Given the significant influence of physical activity on an individual’s health, it is crucial to better understand the factors that influence physical activity levels among children, particularly those who are at most risk of being physically inactive. Current knowledge on the influence of FMS competency on physical activity levels in children from low-income communities is limited. Although previous studies have demonstrated that highly skilled children are significantly more active than children with lower levels of motor skill proficiency during PE lessons [20], little is known regarding the influence of FMS competency on children’s physical activity during recess, lunch and immediately after school. Therefore, the purpose of this study was to examine the associations between FMS competency and objectively measured MVPA during time periods of the day that represent key physical activity opportunities for children (i.e., lunchtime, recess and after-school) among children attending primary schools in low-income communities.

## Methods

### Study design

Baseline data from the Supporting Children’s Outcomes using Rewards, Exercise and Skills (SCORES) group randomized controlled trial was used for the current study. A detailed description of the SCORES study protocol has been published elsewhere [21]. In summary, SCORES was a 12-month multi-component physical activity and FMS intervention for children attending primary schools in low-income communities. Baseline assessments were conducted in February-April 2012 (summer-autumn). Ethics approval for the study was obtained from the Human Research Ethics Committees of the University of Newcastle, Australia and the New South Wales (NSW) Department of Education and Communities. School Principals, teachers, parents and study participants provided written informed consent.

### Setting and participants

Socio-Economic Indexes for Areas (SEIFA) index [22] of relative socioeconomic disadvantage was used to identify eligible primary schools. The SEIFA index (scale 1 = lowest to 10 = highest) summarizes the characteristics of people and households within an area and was developed using the following data: employment, education, financial wellbeing, housing stress, overcrowding, home ownership, family support, family breakdown, family type, lack of wealth (no car or telephone), low income, Indigenous status and foreign birth [22]. Sixteen government primary schools located in the Newcastle region, NSW, Australia with a SEIFA index of ≤5 (lowest 50%) were invited to participate in the study and eight schools (mean SEIFA index of 3 ± 1.3) consented to participate (50% consent rate). All students in grades 3 and 4 (Stage 2) at the study schools were invited to participate in the program. From the 592 eligible children at the eight schools, 460 children consented to participate (78% consent rate).

### Measures

Trained research assistants conducted all assessments, which were completed at the study schools. For consistency and accuracy, a protocol manual, which included specific instructions for conducting all assessments, was developed and used by research assistants to standardize procedures and for quality assurance.

### Physical activity

Physical activity was assessed using triaxial ActiGraph GT3X and GT3X + accelerometers (ActiGraph, LLC, Fort Walton Beach, FL). Output from the vertical axis was used. Vertical axis output from ActiGraph accelerometers appear to be comparable between different generations of ActiGraph accelerometers [23]. Accelerometers were worn by participants during waking hours for seven consecutive days, except while bathing and swimming. Trained research assistants, following standardized accelerometer protocols [24], fitted the monitors and explained the monitoring procedures to students. Data were collected and stored in 10-second epochs with a frequency of 30 Hz. Valid wear time for school-day physical activity (lunchtime, recess and after-school) was defined as a minimum of three weekdays with at least ten hours (600 minutes/day) of total wear time recorded. Valid wear time for total physical activity was defined as a minimum of three days including a weekend day with at least ten hours (600 minutes/day) of total wear time recorded; non-wear time was defined as strings of consecutive zeros equating to 20 minutes [25]. Thresholds for activity counts were used to categorize physical activity into moderate-to-vigorous intensity activity and minutes spent in this activity intensity [26]. The cut-points [26] used were published as 15-second thresholds and were divided by 15 and then multiplied by 10 to create cut-points for 10-second data [27]. Data was further categorized into lunchtime and recess time periods based on individual school timetables. Study schools were contacted at the start of the study and were asked to provide a day-by-day timetable for their recess and lunch breaks. Although we did not conduct direct observations during break times throughout the school day, schools are required by policy to adhere to the timetabled breaks. The lunchtime time period ranged from 45 to 50 minutes in duration and the recess time period was 20 minutes in duration. The after-school time period was defined as the period of time from when school ended for each participant (3.00 pm for all participants) to 6.00 pm.

### Fundamental movement skills

FMS competency was assessed using the Test of Gross Motor Development (TGMD) 2 [28] which has established validity and reliability in children [28]. The TGMD-2 includes six locomotor (i.e., run, gallop, hop, leap, horizontal jump, slide) and six object-control (i.e., striking a stationary ball, stationary dribble, kick, catch, overhand throw, underhand roll) skills. Participants performed each skill twice and skills were video-taped for assessment. Each skill includes several behavioral components. If the participant performed a behavioral component correctly they received a score of 1; if they performed it incorrectly they received a 0. This procedure was completed for each of the two trials, and scores were summed to obtain a total raw skill score. The raw skill scores were then added to obtain a raw locomotor subtest score and a raw object-control subtest score [28]. Inter-rater reliability (98% agreement rate) and intra-rater reliability (97% to 99% agreement rate) were established using pre-coded video tapes before movement skills were assessed by two assessors. Kappa values were also calculated to take into account agreement beyond chance. These were 0.97 for inter-rater reliability and ranged from 0.94 to 0.98 for intra-rater reliability.

### Height and weight

Height was recorded to the nearest 0.1 cm using a portable stadiometer (Model no. PE087, Mentone Educational Centre, Australia). Weight was measured in light clothing without shoes using a portable digital scale (Model no. UC-321PC, A & D Company Ltd, Tokyo Japan) to the nearest 0.1 kg. Body mass index (BMI) was calculated using the standard equation (weight[kg]/height[m]^2^) and BMI-z scores were calculated using the ‘LMS’ method [29].

### Participant demographics

Participating children completed a questionnaire to obtain demographic information including age, sex, language spoken at home, cultural background, Aboriginal or Torres Strait Islander decent, and suburb. The suburb of the child’s residence was used to determine their socio-economic status (SES) using the SEIFA index of relative socioeconomic disadvantage [22].

### Statistical analyses

All analyses were performed using IBM SPSS Statistics for Windows, Version 20 (2011 SPSS Inc., IBM Corp., Armonk, NY). Prior to analyses, normality of the data were assessed and transformed where necessary. Total daily, lunchtime, recess and after-school MVPA minutes were log transformed. Sex differences in demographics, FMS and MVPA measures were tested using unpaired *t-*tests. To assess the cross-sectional associations between FMS (locomotor skills or object-control skills) and MVPA multilevel linear mixed models were used, with MVPA (i.e., total daily, lunchtime, recess or after-school MVPA minutes) as the outcome variable, FMS (i.e., locomotor skills or object-control skills) as the predictor variable, sex, age, BMI-z score and SES as fixed factors (i.e. covariates), and school as a random factor. The mixed models were performed separately for each of the FMS measures (i.e., locomotor skills and object-control skills) and MVPA measures (i.e., total daily, lunchtime, recess and after-school MVPA minutes). The results of the multilevel linear mixed models were also expressed in the form of standardized regression coefficients. These coefficients were computed by initially calculating the mean and standard error for the variable. A new variable was subsequently created by subtracting the mean from the original value, and then dividing the difference by the standard error. The new standardized variables were used in the mixed models regression analyses. This process was performed for all outcome and predictor variables of interest. In all analyses, statistical significance was set at *P* < 0.05.

## Results

### Participant characteristics

The summary data and sex differences for participants’ background, FMS and physical activity are presented in Table 1. Participating children (N = 460, 54% girls) had a mean age of 8.5 ± 0.6 years. Most of the participants (97.6%) reported English as their first language. 86.5% of participants reported Australian as their cultural background, 4.6% as European, 1.3% as African, 0.7% as Asian and 7% as other. 13.8% of participants were of Aboriginal or Torres Strait Islander decent. 22.8% of the sample was overweight and 17% were obese. No sex differences were identified for age, BMI-z score or SES. Participating children’s mean scores for locomotor and object control-skills were 25.7 ± 5.8 and 24.2 ± 6.2 respectively. Girls were more competent on average in locomotor skills (*P* = 0.008), while boys were found to be more competent in object-control skills (*P* < 0.001). Participating children spent an average of 54.8 ± 19.7 minutes per day in MVPA. Compared with girls, boys spent more minutes in MVPA for all time periods. The data indicates that participating children accumulate approximately 50% of their daily MVPA during lunchtime, recess and after-school time periods combined.

**Table 1:** Descriptives and sex differences for children’s background, fundamental movement skills and physical activity

	**All**			**Girls**			**Boys**			**Sex difference**
	***n***	**Mean**	**SD**	***n***	**Mean**	**SD**	***n***	**Mean**	**SD**	***P***
**Background**										
Age (years)	460	8.5	0.6	249	8.5	0.6	211	8.6	0.7	0.123
BMI-z score	458	0.75	1.25	248	0.67	1.22	210	0.84	1.29	0.156
SES^a^	460	3	2	249	3	2	211	3	2	0.879
**FMS (raw)**										
Locomotor skills	430	25.7	5.8	220	26.4	5.2	210	24.9	6.3	0.008*
Object-control skills	456	24.2	6.2	247	21.9	5.3	209	26.8	6.0	0.000*
**Physical activity**										
Total daily MVPA minutes	220	54.8	19.7	125	49.1	17.0	95	61.9	20.6	0.000*
Lunchtime MVPA minutes	325	5.4	3.8	185	4.6	3.3	140	6.5	4.1	0.000*
Recess MVPA minutes	325	5.8	4.4	185	4.8	4.1	140	7.0	4.5	0.000*
After-school MVPA minutes	325	15.5	11.7	185	14.4	13.2	140	16.9	9.3	0.039*

*Abbreviations: FMS*, fundamental movement skills; *MVPA*, moderate-to-vigorous physical activity; BMI, body mass index; SES, socio-economic status.

^a^Socio-Economic Indexes for Areas (SEIFA) index of relative socio-economic disadvantage (1 = most disadvantaged, 10 = least disadvantaged). *Significant difference between girls and boys, *P* < 0.05.

### Locomotor skills and MVPA

After adjustment for sex, age, BMI-z score and SES, locomotor skill competency was positively associated with total (*P* = 0.002) and after-school (*P* = 0.014) MVPA, but not with lunchtime (*P* = 0.075) or recess (*P* = 0.108) MVPA (Table 2).

**Table 2:** Relationships between MVPA minutes and locomotor skills

	**Total Daily MVPA**					**Lunchtime MVPA**					**Recess MVPA**					**After-school MVPA**				
	***n***	**β**	**95% CI**	***r***	***P***	***n***	**β**	**95% CI**	***r***	***P***	***n***	**β**	**95% CI**	***r***	***P***	***n***	**β**	**95% CI**	***r***	***P***
**Locomotor skills**	213	0.010	0.004	0.15	0.002	314	0.010	−0.001	0.08	0.075	314	0.008	−0.002	0.06	0.108	314	0.016	0.003	0.13	0.014
			0.016					0.020					0.018					0.029		
BMI-z score	213	−0.031	−0.057	−0.10	0.017	314	−0.023	−0.068	−0.04	0.303	314	−0.022	−0.065	−0.03	0.306	314	−0.045	−0.099	−0.08	0.103
			−0.006					0.021					0.020					0.009		

*Abbreviations:* MVPA, moderate-to-vigorous physical activity; BMI, body mass index; SES, socio-economic status. All analyses were adjusted for sex, age, BMI-z score and SES and the effects of clustering by school. β values are logged therefore cannot be interpreted in the unit values of the variable.

### Object-control skills and MVPA

After adjustment for sex, age, BMI-z score and SES, object-control skill competency was positively associated with total (*P* < 0.001), lunchtime (*P* = 0.030), recess (*P* = 0.006) and after-school (*P* = 0.022) MVPA (Table 3).

**Table 3:** Relationships between MVPA minutes and object-control skills

	**Total Daily MVPA**					**Lunchtime MVPA**					**Recess MVPA**					**After-school MVPA**				
	***n***	**β**	**95% CI**	***r***	***P***	***n***	**β**	**95% CI**	***r***	***P***	***n***	**β**	**95% CI**	***r***	***P***	***n***	**β**	**95% CI**	***r***	***P***
**Object-control skills**	219	0.013	0.006	0.20	0.000	321	0.012	0.001	0.10	0.030	321	0.015	0.004	0.11	0.006	321	0.015	0.002	0.13	0.022
			0.019					0.023					0.025					0.028		
BMI-z score	219	−0.030	−0.055	−0.09	0.021	321	−0.027	−0.070	−0.04	0.219	321	−0.020	−0.062	−0.03	0.343	321	−0.045	−0.098	−0.08	0.096
			−0.005					0.016					0.022					0.008		

*Abbreviations: MVPA*, moderate-to-vigorous physical activity; *BMI*, body mass index; *SES*, socio-economic status. All analyses were adjusted for sex, age, BMI-z score and SES and the effects of clustering by school. β values are logged therefore cannot be interpreted in the unit values of the variable.

## Discussion

The aim of the current study was to examine the associations between FMS competency and objectively measured MVPA during time periods of the day that represent key physical activity opportunities for children. It was found that object-control skill competency, but not locomotor skill competency was significantly associated with children’s MVPA during lunchtime and recess breaks at school. Children who were more competent at object-control skills and locomotor skills were engaged in more MVPA in the after-school period.

To the authors’ knowledge, this is the first study to investigate the association between children’s FMS competency and MVPA throughout key periods during the school day. Outside of curriculum time, lunchtime and recess periods provide important opportunities for children to be active within the school environment [8]. The current study found that object-control skill competency, but not locomotor skill competency, was significantly associated with MVPA during lunchtime and recess breaks. This finding may be indicative of the games and equipment provided to children during this time period. Games and activities such as soccer and basketball are popular break-time activities which are highly active and require high levels of object-control skill competency [30]. It is possible that the more skilled children dominate these games and the areas available for activity, thus increasing their activity levels and reinforcing the divide between the low skilled and high skilled children.

The school environment and existing policies may also influence children’s activity levels during recess and lunch breaks [31]. Ridgers and colleagues [31] found that overall facility provision (i.e., sum of facilities available) and the provision of unfixed equipment, such as loose equipment, balls, skipping ropes, contribute to increased levels of physical activity among children during school break times. Providing children with access to a variety of facilities, spaces and equipment may encourage physical activity by increasing feelings of choice and supportive environments that foster physically active behaviors [31]. Interestingly, Ridgers and colleagues [31] found stronger effects for children who were less active at baseline. However, it is possible that such approaches will support the activity levels of more skilled children and fail to engage the least skilled individuals. Further research is needed to explore the impact of such policies on the activity of all students, regardless of their existing skill levels.

The significant contribution that physical education can make in the promotion of lifelong physical activity through the teaching of skills and positive behaviours has been well established [6,32]. Fairclough and colleagues [20] found that the more highly skilled students engaged in more MVPA (approximately 5%) during physical education lessons than the less skilled students. Movement skill competency may affect the degree to which skills are effectively performed, which could impact on the potential level of activity achieved in a physical education activity [20]. Thus, higher skilled children would be expected to be more active than less skilled children. Fairclough and colleagues [20] illustrated the need for teachers to use quality pedagogical strategies during physical education that provide all students with equal opportunities for successful movement skill acquisition and optimal physical activity engagement that can be then transferred outside of lessons. Moreover, it is noted that physical education should not be seen as a solution to overcome the increases in physical inactivity in children. Rather, it should be viewed as a regular educational environment (i.e., opportunity for children to learn movement skills) that complements other physical activity opportunities within the school environment. Physical education combined with other school-based opportunities, such as lunchtime and recess breaks, can make a valuable contribution to children’s daily physical activity [6,20,33].

With increasing time during the after-school period spent indoors using electronic entertainment [34], it is important to identify the determinants of children’s physical activity during this time. The after-school period is a “window of opportunity” for promoting physical activity and has the potential to make a substantial contribution to children’s daily physical activity [35]. The study findings suggest that children who are more competent in object-control and locomotor skills participate in more MVPA during the after-school period. These results are consistent with existing evidence of cross-sectional studies in primary school aged children. Raudsepp and colleagues [36] reported the development level of FMS is associated with skill-specific after-school physical activity, with throwing and jumping skills related to higher intensity, skill-specific physical activity. Although these findings were similar to our study, Raudsepp and colleagues [36] only assessed two FMS (overhand throw and standing long jump) and used an observational tool to assess physical activity.

Although BMI was found to be a significant factor in children’s MVPA, the findings in the current study demonstrate that locomotor skill competency and object-control skill competency was a stronger predictor of children’s MVPA throughout the day than BMI. This adds to existing evidence by Spessato and colleagues [37] who found that overall movement skill competency was a better predictor of physical activity during physical education lessons than BMI. This provides further evidence for the development of FMS competency as a key strategy in the promotion of children’s MVPA.

Developing an understanding of the role of FMS competency in promoting physical activity is an important health priority. It is important to consider the bidirectional relationship between FMS and physical activity i.e., whether higher FMS competency increases a child’s physical activity or whether greater participation in physical activity improves FMS competency. Due to the cross-sectional design of the current study, the direction of this relationship cannot be inferred. There is limited research investigating the potential causal relationships between FMS competency and physical activity behavior. However, Barnett and colleagues [38] found a reciprocal relationship between object-control competency and MVPA, and a one-way relationship from MVPA to locomotor skill competency. Although the explicit development of movement skills appears to be an important focus for increasing children’s physical activity levels, it is also important to consider the impact of movement opportunities. It is suggested that if the relationship between skill competency and physical activity is viewed as a “positive feedback loop”, skill development and increasing physical activity should be simultaneously targeted [38]. This has important implications for school and after-school programs policy and practice. Providing quality teaching of FMS during physical education and sport [6], may be as equally important as ensuring the lunchtime and recess environment is conducive to physically active choices (i.e., providing access to sporting equipment during breaks, and utilizing sports equipment and games that target both locomotor and object-control skill use) in the promotion of children’s physical activity [31].

Recent reviews of the effectiveness of physical activity interventions [39,40] have reported modest findings in the promotion of physical activity. This may be in part due to an inadequate understanding of the unique primary factors that influence physical activity for a particular population i.e., low socio-economic position, in a specific context i.e., lunchtime, recess or after-school. In light of these findings, future physical activity interventions are encouraged to focus on improving children’s FMS, providing physical activity opportunities and environments for skill practice and application during school break-times and after-school.

### Strengths and limitations

The current study has a number of strengths, including the use of a comprehensive qualitative assessment of movement characteristics of all major FMS, an objective measure of physical activity, adjustment of all analyses for confounders, and a relatively large sample size. However, there are some limitations that should be noted. Accelerometers underestimate certain types of physical activity as they cannot be worn in the water and are insensitive to non-ambulatory activity such as cycling. Accelerometer wear time criteria are typically generated from whole-day data, therefore it is uncertain if the same criteria can be applied to discrete segments of the day [41]. Due to the cross-sectional design of this study a cause-and-effect relationship between FMS and physical activity cannot be inferred.

## Conclusions

Findings from the current study suggest that children who are more competent in FMS spend more time engaged in MVPA, in particular during time periods of the day that represent key physical activity opportunities for children. Children who are more competent in object-control skills are engaged in more MVPA during lunchtime and recess breaks at school, and children who demonstrate a higher level of competency in locomotor skills and object-control skills engage in more MVPA after-school. Object-control skill competency appears to be a better predictor of children’s MVPA during school-based physical activity opportunities than locomotor skill competency. This suggests that improving movement skill competency, particularly object-control skills, among children is a potential avenue for promoting children’s MVPA throughout the day. Findings from the current study substantially contribute to the understanding of physical activity behaviors in children and will assist in evidence-based school practice, polices and intervention design to increase physical activity.

## Abbreviations

SCORES: Supporting Children's Outcomes using Rewards, Exercise and Skills; FMS: Fundamental movement skills; PA: Physical activity; MVPA: Moderate-to-vigorous physical activity; BMI: Body mass index; SES: Socio-economic status; SEIFA: Socio-economic indexes for areas; TGMD-2: Test of Gross Motor Development 2.

## Competing interests

The authors declare that they have no competing interests.

## Authors’ contributions

DRL, PJM, RCP and RC designed the SCORES project from which the data were drawn. KEC analyzed the data and drafted the article. DRL assisted with data analysis and interpretation. DRL, PJM, RCP and RC revised the manuscript for important intellectual content. All authors conceptualized and designed the idea for the paper and gave approval for the final version to be published.
